# Exploring the Lipidome: Current Lipid Extraction Techniques for Mass Spectrometry Analysis

**DOI:** 10.3390/metabo10060231

**Published:** 2020-06-03

**Authors:** Julian Aldana, Adriana Romero-Otero, Mónica P. Cala

**Affiliations:** Metabolomics Core Facility–MetCore, Vicepresidency for Research, Universidad de los Andes, Cra 1 No. 18A—12, Bogota 111711, Colombia; j.aldana11@uniandes.edu.co (J.A.); a.romeroo@uniandes.edu.co (A.R.-O.)

**Keywords:** extraction, lipids, untargeted lipidomics, targeted lipidomics

## Abstract

In recent years, high-throughput lipid profiling has contributed to understand the biological, physiological and pathological roles of lipids in living organisms. Across all kingdoms of life, important cell and systemic processes are mediated by lipids including compartmentalization, signaling and energy homeostasis. Despite important advances in liquid chromatography and mass spectrometry, sample extraction procedures remain a bottleneck in lipidomic studies, since the wide structural diversity of lipids imposes a constrain in the type and amount of lipids extracted. Differences in extraction yield across lipid classes can induce a bias on down-stream analysis and outcomes. This review aims to summarize current lipid extraction techniques used for untargeted and targeted studies based on mass spectrometry. Considerations, applications, and limitations of these techniques are discussed when used to extract lipids in complex biological matrices, such as tissues, biofluids, foods, and microorganisms.

## 1. Introduction

**Figure 1:**
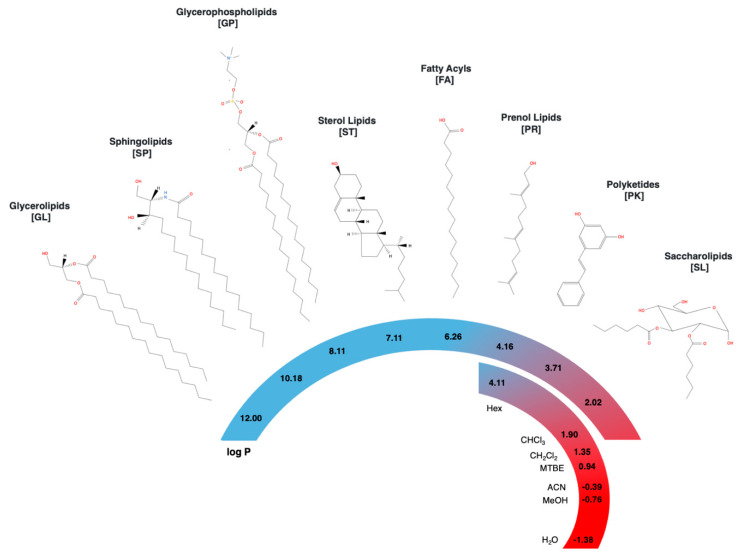
LIPID MAPS categories and representative structures with calculated octanol/water partition coefficient (log P) using ChemAxon. Reported log P of solvents used in lipidomics are indicated below [30]. Color code represents relative polarity, non-polar (blue), and polar (red). Example of classes corresponds to Glycerolipids, DG(16:0/16:0/0:0)—L02010001; Sphingolipids, SP(16:0/16:0)—LMGP01010564; Glicerophospholipids, PC(16:0/16:0)—LMGP01010564; Sterol lipids, Cholesterol—LMST01010001; Fatty acyls, C16:0—LMFA01010001; Prenol lipids, 2E,6E-farnesol—LMPR0103010001; Polyketides, Pinosylvin—LMPK13090001; Saccharolipids, 2,3-di-0-hexanoyl-α-glucopyranose—LMSL05000001.

The term lipids generally refers to amphiphilic organic molecules, poorly soluble in water but miscible in organic solvents. Classification and study of lipid species is challenging due to the large chemical and structural diversity, including different hydrocarbon backbone lengths, branching, unsaturations, and functional groups. Since 2005, the International Lipid Classification and Nomenclature Committee (ILCNC) on the initiative of the Lipid Metabolites and Pathways Strategy (LIPID MAPS) consortium defined lipids as “hydrophobic or amphipathic small molecules that originate entirely or in part by carbanion-based condensations of thioesters and/or by carbocation-based condensations of isoprene units” [1,2,3]. Current lipid classification involves eight categories based on chemical functionalities as: (1) glycerolipids (GL), (2) sphingolipids (SP), (3) glycerophospholipids (GP), (4) sterol lipids (ST), (5) fatty acyls (FA), (6) prenol lipids (PR), (7) polyketides (PK), and (8) saccharolipids (SL), where the last two categories are not synthesized by mammals and represent a small proportion of the known lipidome [1,2,3]. Table 1 presents the number of lipid structures per category according to Lipid Maps^®^ Structure Database (LMSD) and Figure 1 shows representative structures for each category.

Once viewed as mere membranes constituents and energy storage reservoirs, nowadays lipids are also recognized for playing crucial roles in diverse biological activities at cellular and systemic levels including: cell signaling, transport, protein trafficking, growth, differentiation, and apoptosis [3,4]. To accomplish these myriad of functions, cells produce lipids with a vast structural complexity, along with a differentiated compartmentalization, location, organization and interaction [5]. Consequently, a particular set of lipids—known as lipidome—characterize each cell, tissue, and biological system [4].

Lipidomes are often are complex mixtures of lipids, with diverse chemical structures that represent the different biological microenvironments where lipids normally play their function in vivo. Therefore, lipidomes are highly susceptible to changes in response to physiological, pathological, and environmental conditions and can indicate an organism status in a particular moment [6]. In fact, abnormalities in the metabolism of lipids have been linked to several human pathologies (e.g., Alzheimer’s disease [7], cancer [8], diabetes [9]), stress response in plants [10] and antibiotic resistance in infectious bacteria [11,12]. For this reason, the study of lipids has represented a valuable tool to elucidate mechanistic insights into all kingdoms of life.

The main analytical platforms for lipid analyses include mass spectrometry (MS) and nuclear magnetic resonance (NMR), where MS-based techniques have been widely used due to their high sensitivity (pM concentrations), availability and speediness in accurate identification, quantification and monitoring of basal lipid profiles in complex biological mixtures [13]. Sample preparation for MS-lipidomics usually includes solvent–protein precipitation, lipid extraction, and solvent evaporation. The initial step of protein precipitation aims to eliminate matrix components that could interfere with the precision and accuracy of the mass analysis, such as proteins and salts. The subsequent step of lipid extraction takes advantage of the hydrophobic properties of lipids to separate them in a non-polar solvent system with or without mechanical assistance (e.g., vortex, microwave, ultrasound). Finally, solvent evaporation allows lipid enrichment and resuspension in a compatible solvent for MS equipment, typically hyphenated to chromatographic separation [14].

Considering the analytical challenge of extracting hundreds of lipidic compounds with a wide range of polarities and concentration levels, there is not a unique method suitable to extract an entire lipidome. Thus, the choice of a particular lipid-extraction protocol must account for its inherent limitations and be tailored to a specific biological matrix, analysis approach and experimental design. The importance of this choice lies in the profound impact on the class of lipids that can be detectable and measured, which could create a bias on the subsequent analysis and findings. In general, two main approaches are used to analyze lipidomes: (1) non-targeted analysis, also known as hypothesis-generating, to simultaneously extract all detectable lipids in a sample regardless of class, concentration or prior identification, for which non-selective methods are used; (2) targeted analysis, or hypothesis-driven, to selectively extract a particular set of known lipids, in order to avoid interferences and enhance extraction efficiency [15].

Furthermore, repeatability and reproducibility during extraction should account for the lack of analyte-specific internal standards to asses lipid concentrations. In lipidomics, and metabolomics in general, multiple samples are analyzed under equivalent conditions to allow further comparison of analyte levels between different groups (e.g., control vs. disease) using univariate and multivariate statistical analysis. For this reason, biological variations most prevail over analytical and random variations in order to avoid data misinterpretation. In light of the vast diversity of lipids structures and its determining role in lipid extraction, this review summarizes current extraction protocols used for isolation of lipid species present in complex biological matrices, including microorganisms, biofluids, plant and animal tissues, and foods. Technical details concerning both, untargeted and targeted approaches, are discussed along with limitations and considerations of lipid extraction protocols employed in MS-based lipidomics.

## 2. Non-Targeted Analysis

Lipid extraction is without a doubt the major limiting step to analyze the complete set of lipids in biological systems under an untargeted approach. Liquid–liquid extraction (LLE) protocols based on chloroform/methanol mixtures, such as Folch [16] or Bligh and Dyer (BD) [17], have been widely used since the late 1950s with few modifications and still represent the benchmark in the field. Alternative LLE protocols include Methyl tert-butyl ether (MTBE) [18] and Butanol-methanol (BUME) [19] with comparable outcomes for lipid isolation in plasma samples [20,21]. The success of these protocols relies on exploiting the characteristic amphipathic properties of lipids to achieve a differential partition between an aqueous phase and an immiscible organic phase at given temperature, pH and ionic strength. In LLE protocols, separation in two phases is achieved by the hydrophobic and hydrophilic interactions within a system. Hydrophobic interactions are mediated by aliphatic and cyclic hydrocarbon backbones, while hydrophilic ones by polar groups, such as phosphates or carbohydrates. Lipids can also be isolated into one-phase systems, using either a single or a combination of miscible organic solvents for protein precipitation (PPT) and lipid solubilization. Methanol [22], isopropanol [23], and acetonitrile [24] have been proposed as organic systems for untargeted lipidomics.

Solid phase extraction (SPE) is also a feasible option for lipid extraction. It is based on a partition equilibrium involving the adsorption of lipids to a solid phase, which preconcentrates the hydrophobic compounds prior to desorption. Removal of interfering compounds and impurities is achieved by using SPE cartridges, commonly reverse-phase ones. The process comprises of cartridge conditioning, followed by sample loading, cartridge washing and elution. During these stages, aliphatic backbones in lipid structures interact with non-polar stationary phases (e.g., C8, C18) and are retained until an elution solvent is added. Although, SPE protocols are occasionally used for untargeted approaches, they are more typically used for targeted lipidomics. Other strategies for non-targeted lipid extraction are tailored to specific matrices (e.g., plants, cell lines), sampling (e.g., microextraction), and include the use of mechanical assistance like microwaves (MAE), ultrasound (UAE) among others [25]. Reports using solid phase microextraction (SPME) for comprehensive lipid analysis are also worth mentioning [26,27]. SPME employs sorbent-coated rods/fibers where lipids migrate and diffuse directly from a headspace or liquid, to the fiber or rod, eliminating the washing step required on SPE. Then, thermal or solvent desorption is used for lipid elution and analysis by gas chromatography-mass spectrometry (GC-MS) or liquid chromatography-mass spectrometry (LC-MS) respectively [28].

As shown in Figure 1, chemical structures across lipid classes display a large variety of polarities. Encompassing more than 10 units of log P in the octanol–water coefficient as a polarity index, the wide polarity window of lipid species is much wider than any solvent or solvent mixture can cover [15]. In consequence, none of the current protocols is capable to extract all lipid classes simultaneously with high recovery (>80%), and usually the yields of specific compounds are typically sacrificed at the expense of a wider coverage [13]. For instance, chloroform/methanol protocols can extract all lipid classes with recoveries higher than 50%, but present low recoveries for charged and non-polar lipids, like phosphatidic acids (PA) and lysophospholipids (LPA) in plasma [23,29].

Furthermore, the differential recovery across lipid species can interfere with the analysis to different extents. For example, a sub estimation of ST in cerebrospinal fluid is feasible due to its low concentration compared to major constituents FA and SP [31]. In contrast, a similar analysis in plasma would not have a significant impact on ST, considering the its relatively high concentration in that matrix. However, estimation of low-abundant FA could be affected, particularly oxylipins in the pM range [32]. For this reason, it is recommended to explore different extraction protocols if previous reports on the matrix of interest are not available. Crucial factors to be optimized include solvent system and sample-solvent ratio. A good example for method selection and validation can be found in the work of Van Meulebroek et al. [33].

One might tackle the low recovery limitation proposing sequential extraction steps, by using either the same or a complimentary solvent mixture. However, more extraction steps can introduce a higher systematic variability and increase the time of the procedure, which play a crucial factor in large-scale study settings. Furthermore, extraction protocols tend to be minimal in order to preserve sample integrity and content. Non-selective liquid extraction protocols, such as LLE are preferred to extract lipids, since these avoid harsh conditions that can lead to degradation products or cross-products. Nonetheless, there are some concerns about reproducibility of LLE protocols, in particular when MTBE is used given its high volatility [34]. Therefore, single-phase [23,34,35] and even triphasic systems [36] have been proposed to achieve enhanced reproducibility and lipid coverage. Further validation of these methodologies is still required. Table 2 presents a contrast of current lipid extraction protocols used for untargeted MS-based studies of biological matrices. Different parameters were taking into account for the comparison, including coverage, recovery, reproducibility and automatization. Note that lipidomic studies using matrix-assisted laser desorption-ionization (MALDI) as ionization source were not included in this review (recently reviewed by Leopold et al. [37]), since little or no sample preparation is needed.

In recent years, automatized lipid extraction has been proposed to decrease the experimental variability between samples in large batches. Not all solvent–extraction systems are suitable for this task, since some of them are prone to contamination with proteins and other matrix components. This is the case of chloroform/methanol protocols, where in order to get access to the non-polar fraction at the bottom, the injection needle must break through a protein interphase and a polar fraction at the top. In contrast, MTBE and BUME methods use a low-density extraction solvent that locates the hydrophobic fraction at the top of the partition system, there it can be easily sampled by an automatized needle. Moreover, solvent compatibility with the MS system should be considered. For instance, chloroform is well-known for being problematic with liquid chromatography (LC)-MS and it has to be removed by a previous injection. One-phase extraction (OPE) and SPE protocols could also be automatized for high throughput analysis. A recent review by Liu et al. covers the details and advances in analytical methods for MS-based large-scale lipidomics [73].

Another consideration is the high sensitivity of MS instruments, which could play a double-edged sword role in the mass-to-charge analysis of lipid extracts. On the one hand, small sample amounts are enough to detect femtomolar and quantify picomolar concentrations of lipid species. Usually only 10–100 µL of liquid sample or 1–100 mg of solid sample is required for lipid extraction. Then, 5–20 µL of extract are subsequently taken for either LC injection (LC-MS based lipidomics) or direct infusion into an MS (also called shotgun lipidomics). On the other hand, multi-sourced trace impurities coming from biological matrices (e.g., remaining proteins), solvents, preparation devices (e.g., siloxenes and phthalates), and even sample containers (e.g., plasticizers) can also be detected if carried over in the lipid extract.

Overall, the presence of contaminants excerpts an effect on ionization (either enhancement or suppression) and can lead to deterioration of MS instrumentation. This issue is not as critical in LC-MS methods as in shotgun lipidomics, since LC allows separation between lipids and contaminants. Consequently, HPLC (high-performance liquid chromatography) and UPLC (ultra-performance liquid chromatography) are commonly preferred for non-directed lipidomics at the expense of increased solvent consumption and analysis time. Recently, SFC (supercritical fluid chromatography) have been successfully applied as a sample separation step to overcome these drawbacks [74].

Although, a comprehensive discussion of sampling and sample processing falls outside the scope of this review, general guidelines for handling biological matrices are implemented to preserve lipidome integrity. These measures include sample storage at −80 °C, avoid freeze–thaw cycling, and short processing times at 4 °C to minimize unwanted enzymatic and chemical processes. However, some lipid species (e.g., oxylipins, polyphenols, lysoGP) require particular measures due to their susceptibility to oxidation and isomerization. For this reason, antioxidants such as, butylated hydroxytoluene (BHT), butylated hydroxyanisole (BHA), or triphenylphosphonium (TPP), along with buffers are commonly included prior sample homogenization and extraction. Additionally, the use of MS grade solvents, glass vials and glass pipetting tips significantly decrease the incorporation of contaminants.

Another important limitation of any current extraction protocol is the inherent homogenization of lipids coming from different sub-compartments (e.g., tissue region, cell type, organelles). The disruption of in vivo interactions and subsequent reorganization of lipids by hydrophobicity not only hinders the chance to localize and monitor its dynamic changes, but also can impact its stability and reactivity [13]. Therefore, conclusions from these studies most acknowledge this limitation in the biological interpretation of their outcomes.

Overall, lipidomic studies can be powerful hypothesis-generating tools. Extraction conditions should be adjusted and validated for the biological matrix of interest. General guidelines include testing of repeatability, reproducibility, and recovery using at least one representative compound per lipid class. The step by step workflow for protocol selection proposed by Furse et al. [75] can serve as a starting point.

## 3. Targeted Analysis

For studies focused on a specific subset of lipids, the particular features in their chemical structures drive the selection of the extraction protocol. Isolation and concentration of lipids of interest is achieved by either LLE with narrow polarity windows or SPE cartridges. Compared with untargeted approaches, targeted analysis allows higher sample amounts due to the selective procedures used for enrichment of a particular lipid species. For instance, to study low-abundant oxylipins in plasma, up to 250 µL of the sample are extracted, which represents ten times the typical amount used for untargeted approaches [14].

In general, compounds with a high hydrophobicity index, such as triacylglycerols (TG), diacylglycerols (DG), cholesteryl esters and fatty esters, are commonly extracted with LLE. Non-polar solvents (e.g., cyclohexane, toluene) are preferred over moderately polar solvent mixtures (e.g., chloroform, MTBE) since only the most hydrophobic lipids dissolve in them [75].

The extraction of intermediate hydrophilic species, such as GP and SP, is usually achieved by LLE using polar solvents. Chloroform-based protocols are the most common, followed by a rising popularity of MBTE-based ones. Considering the recovery disparities across lipid species, these protocols are often modified to achieve higher extraction yields, including changes in solvent system, solvent proportions (%v/v), and introduction of mechanic forces. When charged groups are present, another important variable is the pH, since the acid-base character of certain lipids can be used for its extraction and enrichment. For instance, phosphatidic acids (PA), phosphatidylserines (PS) [76] and phosphatidylinositols (PI) [77] can be efficiently extracted using mild acidic conditions. However, pH changes must be optimized to avoid structural re-arrangements in the presence of nucleophiles, given the high electrophilicity of phosphate groups [78].

Finally, isolation of polar lipids has less standardized protocols given both, the similarities in physicochemical properties to common non-lipid metabolites and the complex structure of these compounds (e.g., acylaminosugars, cerebrosides). Typical approaches use water, methanol, or pyrimide as polar solvents in combination with additives [75]. When SPME is used, derivatization reagents are incorporated into the SPME fibers to improve specificity and sensitivity. Table 3 provides a revision of recent lipidomic targeted studies in different biological matrices. A discussion for each lipid category is also presented.

### 3.1. Glycerolipids (GL)

GL are neutral lipids composed of one glycerol backbone attached to at least one FA via ether or ester linkage. Structural classification is mainly based on the number of FA bound. Mono-, di-, and triradylglycerols are typically extracted with apolar organic solvents, including octane, cyclohexane, and MTBE. An alternative approach includes supercritical CO_2_ for high-throughput separation of GL [165]. Pure chloroform and mixtures with methanol have also been reported [84,104,106,108]. Selective isolation of these hydrophobic species is achieved with low degradation, under mild conditions used in the extraction and minor reactivity of GL. Glycosylglycerols are another important subgroup in this class, which are characterized by sugar residues linked to the backbone. BD and Folch methods have been reported for the analysis of glycosylglycerols, such as digalactosyldiacylglycerols [110] and seminolipids [166].

### 3.2. Sphingolipids (SP)

The common backbone in SP consists of an amino alcohol, known as sphingoid base. Structural diversity arises from different chemical moieties linked to the amino and hydroxy groups. Important members of this family include ceramides, phosphospingolipids, cerebosides, and gangliosides. An up-to-date review by Montefusco et al. summarizes several experimental considerations for quantitative and qualitative analysis of sphingolipids [167]. In general, reports are consistent in comparable SP recoveries using chloroform-methanol-based and MTBE methods, with electrospray (ESI) as ionization source in positive and negative modes [21,23,62]. Mild alkaline and acidic extraction has also been combined with classic LLE protocols. Addition of MeONa [125] and KOH [126] has shown improved recovery and reproducibility in SP extraction, particularly ceramides and phosphosphingolipids. A standardized protocol for SP profiling is proposed by Sullards et al. [126]. For gangliosides, a quantitative approach involves derivatization using 4-(4, 6-Dimethoxy-1, 3, 5-triazin-2-yl)-4-methylmorpholinium chloride (DMTMM) and 2-(2-Pyridilamino)-ethylamine (PAEA) after protein precipitation [131]. The addition of a pyridylamine group increases the ionization efficiency resulting in a 15-fold signal intensity when compared to previously reported methods.

### 3.3. Glycerophospholipids (GP)

GP basic structure contains a glycerol base linked to one or two FA at the sn-1 and sn-2 positions. A phosphate group is also attached to the sn-3 position. Different bonding (acyl-, alkyl-, or alkenyl), length and unsaturation of the FAs create a wide range of combinations. However, GP are commonly subclassified based on the head group attached to the phosphate. Neutral phosphatidylcholine (PC) and phosphatidylethanolamine (PE) species, and charged ones including phosphatidic acid (PA), phosphatidylglycerol (PG), phosphatidylinositol (PI), phosphatidylserine (PS) and cardiolipins (CL) have precise locations and functions within cell membranes [168]. Most of neutral GP are successfully extracted with traditional procedures using chloroform or MTBE with comparable yields, except for LPCs [21,169]. For polar GPs like PI and LPA acidic conditions (pH 4–6) during extraction are recommended [170]. Tris or citric acid buffers are preferred for this purpose to avoid hydrolysis and interconversion of GP [119]. OPE protocols have also shown improved recoveries in polar GP, particularly PG and PS [23]. As alternative, specific SPE cartridges are commercially available for deproteinization and phospholipid preconcentration, such as HybridSPE-Phospholipid Ultra cartridge^®^ [171] and iSPE^®^, with reported capability to isolate low-abundant GP [169,172,173].

### 3.4. Sterol Lipids (ST)

The structural skeleton of ST is based on a four fused ring. Differences in conjugation and position/type of polar functional groups generate a broad spectrum of polarities in this family. Lipid analysis in mammalian systems have focused on steroids, cholesterol and bile acids, while plants systems focus on phytosterols and phytostanols. Thin layer chromatography–flame ionization (TLC-FID) and GC-MS techniques were initially used to determine ST with the requirement of derivatization (e.g., trimethylsilation and methylation). Nonetheless, an increasing number of publications have been implemented LC-MS strategies, since ST analysis can be performed in native state. Commonly, PPT or LLE methods are used for lipid extraction prior SPE in order to avoid interferences and signal suppression. Appropriate SPE elution solvents can resolve cholesterol from derivates like oxysterols and steroids given the differences in number of hydroxyl groups attached to the core ring. Moreover, antioxidants and metal chelators are commonly added to prevent ST oxidation during the extraction procedure [174]. For ST in plants, n-hexane and supercritical CO_2_ have shown the best yields when combined with mechanical forces [175]. If esterified ST are targeted, a saponification stage under mild conditions can be included to remove fatty acyl groups without generation of artifacts [176].

### 3.5. Fatty Acyls (FA)

The structure of FA contains repeated series of methylene groups, derived from successive additions of malonyl-CoA or methylmalonyl-CoA to an acetyl-CoA primer. Differences in carbon chain lengths, degree of oxidation, unsaturation, and cyclations are the main sources of structural diversity. Major subclasses include fatty acids, fatty esters, and eicosanoids. Fatty acids have been traditionally derivatized for characterization by GC–MS. However, LC-MS and SFC-MS approaches provide quantitative signals for most fatty acids in biological samples. This allows the use of faster and milder conditions during lipid extraction, like BD and MTBE protocols. For extraction of eicosanoids, further measurements must be taken to avoid lipid degradation and artifact generation. Cold conditions (4 °C) and addition of antioxidants (e.g., BHT, BHA) are commonly introduced to preserve FA integrity within a sample. In a recent review on oxylipin extraction, Liakh et al. contrast PPT, LLE, and SPE methods. A combination of LLE previous SPE seems to be the best approach for preconcentration of oxylipins and other related species [177].

### 3.6. Prenol Lipids (PR)

PR carbon backbones comprise one or more isoprene units condensed. The number of terpene units and oxidation state of the structure form the basis of their classification. Isoprenoids, quinones and polyprenols are representative members of this class. Besides the well-known derivatization for GC-MS analysis of PR [178], recent OPE and LLE have been introduced for LC-MS methods. One-phase solvent systems containing diethyl ether, methanol, propanol and water are generally employed for the extraction, due to the relative polar character of PR in comparison to other bulk lipid classes. For higher yields of apolar PR like carotenoinds and tocopherols, either hexane or chloroform is added [148,179]. Supercritical fluids have also shown potential to efficiently extract and separate these compounds as reported in food and human serum studies [180]. Given their physicochemical similarities, ST are commonly co-extracted with PR, even after saponification. However, conventional ST precipitation with petroleum ether [181] or SPE enrichment [151] could be used to achieve separation of PRs. Additionally, extraction conditions such as neutral or slightly basic pH, presence of antioxidants and absence of light improve extraction yield and minimize PR degradation.

### 3.7. Polyketides (PK)

PK are a structural diverse lipid class derived from sequential condensations of ketoacyl groups. PK are characterized by backbones with at least two carbonyls linked by a carbon atom, and also subject to functional modifications including hydroxylation, glycosylation, methylation among others. Further subclassification include macrolides, aromatic polyketides, and flavonoids. The abundance of polar groups and double bounds in PK structures allow the use of polar solvents to narrow down the type of lipids to be extracted. Targeted studies report either ethyl acetate or methanol as OPE solvents. Considering that plants, fungi, and bacteria are the main biological sources of PK, mechanical forces are commonly applied during the extraction. Pressurized systems with [182] or without [183] supercritical fluids increase significantly the PK recovery. SPE methods have also been adopted for PK fractionation and preconcentration using reverse phase cartridges (e.g., Strata™-X(Torrance, CA 90501-1430, USA), Sep-Pak Plus C18), particularly for prymnesins [163] and flavonoids [184]. Other important parameters must be optimized for the extraction, including sample amount, sample-solvent ratio and temperature. Finally, extraction of PK prone to oxidation (e.g., polyphenols) can incorporate β-mercaptoethanol or K_2_S_2_O_5_ to decrease unwanted redox processes.

### 3.8. Saccharolipids (SL)

SL are distinguished by a sugar backbone linked to FAs via glycosidic bonds. Representative members of this class include lipopolysaccharides (LPS) synthesized by gram-negative bacteria. Several methods have been developed for LPS extraction based on the analytical technique for detection and quantification. For MS studies, phenol-based methods are the standard for SL extraction, including hot phenol extraction (HPE), aqueous phenol-chloroform and aqueous phenol-diethyl ether extraction [185]. Recently, alternative at-room-temperature protocols have been tested to improve efficiency, yield, and lipid integrity. SPE [156] and methanol-chloroform [155] methods are reported in cell membrane characterizations.

SL are distinguished by a sugar backbone linked to FAs via glycosidic bonds. Representative members of this class include lipopolysaccharides (LPS) synthesized by gram-negative bacteria. Several methods have been developed for LPS extraction based on the analytical technique for detection and quantification. For MS studies, phenol-based methods are the standard for SL extraction, including hot phenol extraction (HPE), aqueous phenol-chloroform and aqueous phenol-diethyl ether extraction [185]. Recently, alternative at-room-temperature protocols have been tested to improve efficiency, yield, and lipid integrity. SPE [156] and methanol-chloroform [155] methods are reported in cell membrane characterizations.

## 4. Conclusions and Future Perspectives

The high-throughput analysis of lipids using mass spectrometry provides a snapshot into complex lipidomes in living organisms. However, lipid identification and quantification are limited by the selected extraction protocol. Structural features not only drive the intermolecular interactions within an extraction system, but also dictates their stability and reactivity. Furthermore, type and amount of biological matrix should be well considered for protocol selection and validation. Despite the efforts of several researchers, the lack of standardized procedures for sample preparation in lipidomics is still a major concern in the field. Therefore, comparative studies contrasting two or more different isolations techniques, along with inclusion of extraction protocols in lipid databases, provide valuable insights and criterion.

Preservation of lipidome integrity must be prioritized through extraction procedures, otherwise lipid extracts would not faithfully retain biological variations and comparisons across samples will be inaccurate. Automatized lipid isolation represents a feasible solution by taking advantage of simple and versatile protocols. Although not all samples are suitable subjects of automatization, advances in SPME-LC online interfaces and combined extraction of amphiphilic and lipophilic compounds in glass-coated microplates are promising.

For untargeted approaches, the main limitations are the narrow coverage and differential yield of extracted lipid species. Meanwhile, repeatability and reproducibility through several extraction steps are constraints faced by targeted approaches. Future lipidomic studies will account for lipid sub-cellular localization, interaction with physiological partners and monitor concentrations changes over time using fast, reproducible, and versatile extraction protocols to assure an accurate depiction of the dynamic changes taking place in biological systems.

## Figures and Tables

**Table 1:** Number of lipids structures per representative lipid category.

Lipid Category	Main Subclasses	Log P Range ^a^	LIPID Maps ^b^		
			Curated	Computationally-Generated	All
Fatty Acyls [FA]	Fatty Acids and Conjugates, Eicosanoids, Docosanoids, Fatty esters, Fatty amides, Fatty nitriles, Fatty ethers, Fatty acyl glycosides, Acylcarnitines.	−5–15	7644	1792	9436	
Glycerolipids [GL]	Monoradylglycerols, Diradylglycerols, Triradylglycerols, Glycosylmonoradylglycerols, Glycosyldiradylglycerols.	5–35	232	7379	7611	
Glycerophospholipids [GP]	Glycerophosphocholines, Glycerophosphoethanolamines, Glycerophosphoserines, Glycerophosphoglycerols, Glycerophosphoglycerophosphates, Glycerophosphoinositols, Oxidized glycerophospholipids, Cardiolipins.	5–25	1607	8312	9919	
Sphingolipids [SP]	Sphingoid bases, Ceramides, Phosphosphingolipids, Neutral glycosphingolipids, Acidic glycosphingolipids, Basic glycosphingolipids.	5–25	1410	3176	4586	
Sterol lipids [ST]	Sterols, Steroids, Secosteroids, Bile acids and derivatives, Steroid conjugates.	0–20	2829		2829	
Prenol lipids [PR]	Isoprenoids, Quinones and hydroquinones, Polyprenols.	0–20	1352		1352	
Sacccharolipids [SL]	Acylaminosugars, Acylaminosugar glycans, Acyltrehaloses.	0–30	22	1294	1316	
Polyketides [PK]	Linear polyketides, Macrolides and lactone polyketides, Linear tetracyclines, Polyether antibiotics, Aflatoxins, Flavonoids, Aromatic polyketides.	0–15	6810		6810	
TOTAL			21,906	21,953	43,859	

^a^ Octanol/water partition coefficient (log P) calculated using ChemAxon. ^b^ Data taken from Lipid Maps^®^ Structure Database (LMSD) in the 05/02/2020 update.

**Table 2:** Comparison between extraction methods used in mass spectrometry (MS)-based untargeted lipidomics.

Extraction Method	Principle	Protocol Name	System	Lipid Coverage	Lipid Recovery	Solvent-Efficiency	Carry-Over Free	Time-Efficiency	Repeatability	Versatility	Automatization	Biological Matrix	Platform	Ref.
PPT	Precipitation	OPE	*i*PrOH, ButOH/MeOH (2:2) CHCl_3_/MeOH (2:2)									Plasma	HPLC-ESI-QTRAP	[38,39,40]
												CSF	UHPLC-ESI-QTOF	[41]
LLE	Partition	Folch *	CHCl_3_/MeOH (2:1)									Cell line/tissue	UHPLC-ESI-Q-Exactive Orbitrap	[42]
												CSF	HPLC-ESI-Q-Exactive Orbitrap	[43]
		Folch * BD *	CHCl_3_/MeOH (2:1) CHCl_3_ or CH_2_Cl_2_/MeOH (1:2)									Plasma/CSF Food	HPLC-ESI-Ion trap	[44]
													UHPLC-Q-Exactive Orbitrap	[45,46]
												Tears	UHPLC-TripleTOF	[47]
												Plants	HPLC-ESI-QTOF	[48]
												Urine	UHPLC-ESI-3D-Ion trap	[49]
												Feces	HPLC-ESI-Q-Exactive Orbitrap	[50]
												Cell culture	HPLC-ESI-Q-Exactive Orbitrap	[43]
		BD * MTBE *	CHCl_3_ or CH_2_Cl_2_/MeOH (1:2) MTBE/MeOH (5:1.5)									Animal tissue	DI-ESI-QTRAP	[51]
												Urine/saliva	ASAP-QTOF	[52]
												Plant tissue	UHPLC-ESI-IT-TOF	[53,54]
												Feces	HPLC-ESI-Q-Exactive Orbitrap	[50]
												Plasma	HPLC-ESI-QTOF	[55]
		MTBE * BUME	MTBE/MeOH (5:1.5) 1. ButOH/MeOH (3:1) 2. Hep/EtOAc (3:1) 3. 1% AcOH									Urine	HPLC-ESI-QTOF	[56]
												Food	UHPLC-ESI-TOF	[57]
												Breast Milk	HPLC-ESI-QTOF	[58]
												Plant tissue	UHPLC-ESI-Q-Exactive Orbitrap	[59]
												Cell line	HPLC-LTQ-Orbitrap	[60]
												Plant tissue	UHPLC-ESI-Q-Exactive Orbitrap	[61]
												Cell line Plasma/SBF Animal tissue	UHPLC-ESI-QTOF	[62]
													HPLC-ESI-QqQ	[63]
													DI-ESI-QTRAP	[51]
		BUME IPA/Hex *	1. ButOH/MeOH (3:1) 2. Hep/EtOAc (3:1) 3. 1% AcOH*i*PrOH/Hex (3:2)									Cell lines	UHPLC-ESI-Q-Exactive Orbitrap	[64]
												Cell lines	UHPLC-ESI-Q-Exactive Orbitrap	[61]
												Cell lines	UHPLC-ESI-QTOF	[62]
		IPA/Hex * SFE	*i*PrOH/Hex (3:2) Supercritical CO_2_									Serum Parasites	DI-ESI-TOF	[65]
													HPLC-ESI-QqQ	[66]
												Plasma	SFE-ESI-QTRAP	[67]
												Parasites	UHPSFC-ESI-IT-TOF	[68]
		SPE	Coated fibers and capillary tubes (sorbent cartridge).									Animal tissue Plasma	UHPSFC-ESI-IT-TOF	[69]
													HPLC-ESI-Q-Exactive Orbitrap	[70]
SPE	Adsorbance	SPE SPME	Coated fibers and capillary tubes (sorbent cartridge). Diameter-reduced sorbent-coated rods/fibers									Saliva	HPLC-ESI-Q-Exactive Orbitrap	[71]
												Breast Milk	HPLC-ESI-QTOF	[72]
												Cell lines	HPLC-ESI-Q-Exactive Orbitrap	[43]
												Urine	GC-EI-Q	[26,27]

* Water is added for phase separation. OPE: One-phase extraction; PPT: Protein precipitation; BD: Bligh and Dyer; SFE: Supercritical fluid extraction; SPE: Solid-phase extraction; SPME: Solid-phase microextraction; BUME: Butanol-methanol; MTBE: Methyl tert-butyl ether; DI: Direct infusion; ASAP: Atmospheric Solids Analysis Probe; Color reference: Dark blue: Good; Blue: Fair; Light blue: Poor. References were selected for studies conducted during the 2009–2019 period.

**Table 3:** Reports of targeted extraction of lipids by category.

Lipid Class	Extraction Method	System	Biological Matrix (Sample Amount)	Platform	Ref
**Fatty Acyls**					
***Fatty Acids and Conjugates***					
	1. LLE, 2. SPE	1. Modified Bligh and Dyer, CH_3_Cl - MeOH - H_2_O (1:1:1) with 2 mM HCl; 2. 3-aminopropyl silica gel/ACN-AcOH (49:1)	Food supplements (3 mL)	UHPLC-ESI-QTOF	[79]
	LLE	H_2_O with 0.5 M HCl - anhydrous Et_2_O (1:1) + Deriv. with BSTFA	Feces (30 mg)	GC-Q	[80]
	LLE	Modified Folch, CH_3_Cl-MeOH-H_2_O (2:1:0.8) + BHT	Grapes-skins-seeds (0.1 g)	UHPLC-ESI-QqQ	[81]
	LLE	Modified Folch, CH_3_Cl - MeOH - 0.73% NaCl (2:1:0.6) + Deriv. with 1% H_2_SO_4_ in MeOH	Green coffee (0.5 g)	GC-Q	[82]
	LLE	Hex - 0.5% NaOH in MEOH and 14% BF_3_ in MeOH - Sat. NaCl (1.3:2:4)	Shark liver oil (25 mg)	GC-Q	[83]
	1. Filter pre-conc. 2. LLE	1. Whatman GF/F filters, 2. CH_2_Cl_2_ - MeOH - H_2_O (1.5:3:1)	Oceanic water (5–10 L)	DI-ESI- FT-ICR MS	[84]
***Octadecanoids***					
	1. OPE, 2. SPE	1. MeOH + BHT; 2. < 8% MeOH/Polymeric RP/MeOH + BHT	Mice amygdala tissue	UHPLC-ESI-QTRAP	[85]
***Eicosanoids***					
	1. LLE, 2. Hydrolysis, 3. SPE	1. MTBE-MeOH-0.15 M NH_4_OAc (2:1:1) + BHT; 2. MeOH-H_2_O-10M NaOH (1:1:1.2); 3. Reac.Mix. + AcOH + 0.1 M Na_2_HPO_4_ buffer (pH 6)/AEC/tOAc-Hex (75:25) + 1%AcOH	Human cells (1 × 10^7^)	HPLC-ESI-QTRAP	[86]
	LLE	Sample + AcOH (pH 3.5) - MTBE (2:1) + Deriv. for GC	Breath condensate (2 mL)	HPLC-ESI-QTRAP GC/NICI–MS	[87]
	SPE	MeOH (<17%) + Na_2_HPO_4_ buffer (pH 6)/AEC/EtOAc - Hex (75:25) with 1% AcOH	Plasma (500 µL)	UHPLC-ESI-QTRAP	[86]
	SPE	H_2_O + BHT/Polymeric RP/MeOH	Plasma (500 µL)	UPLC-ESI-QqQ	[88]
	SPE	15% MeOH + 0.1 M HCl (pH 3.0)/C18/Methyl formate	Mouse brain tissue (25–75 mg)	HPLC-ESI-QqQ	[89,90]
	SPE	10% MeOH/Polymeric RP/MeOH	Human cells-animal tissues	HPLC-ESI-QTRAP	[91]
	1. OPE, 2. SPE	1. MeOH + BHT; 2. < 8% MeOH/Polymeric RP/MeOH + BHT	Mice brain tissue	UHPLC-ESI-QTRAP	[85]
	SPE	0.12 M PP buffer + 5 mM MgCl_2_ + BHT/Polymeric RP/MeOH	Human CSF and rat cortex	UPLC-ESI-QqQ	[92]
***Fatty Esters***					
	OPE	MeOH - 0.1% FA (0.8:1)	Plasma (20 µL)-urine (5 µL)-CSF (20 µL)	UHPLC-HESI-QTRAP	[93]
	1. OPE, 2. OPE	1. MeOH - H_2_O (4:1) + 0.1% FA; 2. MeOH - 0.1% FA (0.8:1)	Human brain tissue (10 mg)	UHPLC-HESI-Q-Orbitrap	[93]
	OPE	*i*PrOH	Serum (10 µL)	HPLC-ESI-QqQ	[94]
	OPE	AbsoluteIDQ p180 Kit	Serum (10 µL)	HPLC-ESI-QqQ	[95]
	OPE	AbsoluteIDQ p180 Kit	Plasma	UPLC-ESI-QqQ	[96]
	OPE	ACN + Deriv. with DnsHz	Plasma	UHPLC-ESI-QqQ	[97]
	1. LLE, 2. SPE	1. CHCl_3_ - MeOH - PBS (2:1:1); 2./CHCl_3_/Silica NP/EtOAc	Adipose tissue (150 mg)	HPLC-ESI-QqQ	[98]
	1. LLE, 2. SPE	1. CHCl_3_ - MeOH - PBS (2:1:0.86); 2./CHCl_3_/Silica NP/EtOAc	Serum or plasma (200 µL)	HPLC-ESI-QqQ	[98]
	1. LLE, 2. SPE	1. Bligh and Dyer, CHCl_3_-MeOH - H_2_O (2:1:1); 2. 0.1% NH_4_OH in ACN/AEC/1% FA in Acetone + AMPP	Plant leaves (100 mg)	UHPLC-ESI-QqQ	[99]
	SPE	0.1% NH_4_OH in ACN (1:2)/AEC/1% FA in Acetone + Deriv. DMED and d4-DMED	Mice adipose tissue (100 mg)	UHPLC-ESI-QqQ	[100]
	1. LLE, 2. SPE	1. Modified Bligh and Dyer, CHCl_3_ - MeOH (1:1); 2. CHCl_3_/Silica NP/EtOAc	Plasma (100 µL), liver	nanoESI-QqQ	[101]
***Fatty amides***					
	SPE	10% MeOH/Polymeric RP/MeOH	Human cell lines or animal tissues	HPLC-ESI-QTRAP	[91]
	SPE	MeOH - H_2_O (1:2.3)/C18/MeOH	Rat brain	HPLC-ESI-QqQ	[102]
**Glycerolipids**					
***Monoradylglycerols***					
	OPE	MeOH + Deriv. with d_4_-NPB and 3-NPB	Cells-Tissue (60 mg)-Serum (50 µL)	UPLC-ESI-QTOF	[103]
	OPE	*i*PrOH	Shark liver oil (10 mg)	HPLC-ESI-QqQ	[83]
	1. Filter pre-conc. 2. LLE	1. Whatman GF/F filters, 2. CH_2_Cl_2_ - MeOH - H_2_O (1.5:3:1)	Oceanic water (5–10 L)	DI-ESI- FT-ICR MS	[84]
	LLE	CHCl_3_ - MeOH - 25 M LiCl (1:1:1)	Plasma (25 µL)	DI-ESI-QTOF	[104]
***Diradylglycerols***					
	1. OPE, 2. LLE	1. 70% *i*PrOH, 2. Bligh and Dyer	Feces (2 mg)	FIA-ESI-Q-Orbitrap	[105]
	OPE	*i*PrOH	Shark liver oil (10 mg)	HPLC-ESI-QqQ	[83]
	LLE	Modified Bligh and Dyer, CHCl_3_ - MeOH - H_2_O (1:1:0.8)	Bacterial cells (OD: 0.3, 0.5, 0.8, 1.3)	HPLC-ESI-LTQ-Orbitrap	[106]
	LLE	Modified Matyash, MTBE - MeOH - H_2_O (3:0.9:0.75)	Skeletal muscle (50 mg)	HPLC-API-QqQ	[107]
	1. Filter pre-conc. 2. LLE	1. Whatman GF/F filters, 2. CH_2_Cl_2_ - MeOH - H_2_O (1.5:3:1)	Oceanic water (5–10 L)	DI-ESI- FT-ICR MS	[84]
***Triradylglycerols***					
	LLE	Modified Bligh and Dyer, CH_2_Cl_2_ - MeOH - H_2_O (1.5:3:1)	Serum (30 µL)	HPLC-HESI-Orbitrap	[108]
	OPE	EtOH - MTBE - DCM (70:15:15)	Size-fractionated serum (20 µL)	UPLC-ESI-QTRAP	[35]
	LLE	Modified Bligh and Dyer, CHCl_3_ - MeOH - H_2_O (1:1:0.8)	Bacterial cells (OD: 0.3, 0.5, 0.8, 1.3)	HPLC-ESI-LTQ-Orbitrap	[106]
	OPE	Acetone	Vegetal Oil (40 mg)	HPLC-ESI-Quadrupole	[109]
	1. Filter pre-conc. 2. LLE	1. Whatman GF/F filters, 2. CH_2_Cl_2_ - MeOH - H_2_O (1.5:3:1)	Oceanic water (5–10 L)	DI-ESI- FT-ICR MS	[84]
	1. OPE, 2. LLE	1. 70% *i*PrOH, 2. Bligh and Dyer	Feces (2 mg)	FIA-ESI-Q-Orbitrap	[105]
***Glycosyldiradylglycerols***					
	1. pre-treat, 2. LLE	1. *i*PrOH (75 °C) + BHT; 2. CHCl_3_ - MeOH (2:1)	Vegetal tissue (2–3 plant rosettes)	ESI-QqQ	[110]
	LLE	Bligh and Dyer	Algae tissue (30 L culture)	UPLC-ESI-QTOF	[111]
**Glycerophospholipids**					
***Glycerophosphocholines***					
	OPE	AbsoluteIDQ p150 Kit	Plasma (10 µL)	FIA-ESI-QTRAP	[112]
	OPE	ACN	Plasma (20 µL)	UPLC-ESI-QTRAP	[113]
	OPE	MeOH + BHT	Plasma (10 µL)	HPLC-ESI-QTRAP	[114]
	LLE	CH_2_Cl_2_ - MeOH (2:1)	Cytosol (100 µL)	UPLC-ESI-QTRAP	[115]
	LLE	Modified Folch, CHCl_3_ - MeOH - 0.15 M NaCl (2:1:0.8) + BHT	Lenses	ESI-QqQ	[116]
	LLE	Modified Bligh and Dyer, CHCl_3_ - MeOH - 0.1N HCl (1:1:1)	Human cells (2 × 10^6^)	UPLC-ESI-QTOF	[117,118]
	µChip-SPE	Lysozyme in 20 mM Tris-HCl (pH 7.5)/Silica beads/MeOH	Bacterial cells (10µL)	nanoESI-QTOF	[119]
	OPE	EtOH - MTBE - DCM (7:1.5:1.5)	Size-fractionated serum (20 µL)	UPLC-ESI-QTRAP	[35]
	LLE	Bligh and Dyer	Algae tissue (30 L culture)	UPLC-ESI-QTOF	[111]
***Glycerophosphoethanolamines***					
	OPE	ACN	Plasma (20 µL)	UPLC-ESI-QTRAP	[113]
	OPE	MeOH + BHT	Plasma (20 µL)	HPLC-ESI-QTRAP	[114]
	LLE	CH_2_Cl_2_ - MeOH (2:1)	Cytosol (100 µL)	UPLC-ESI-QTRAP	[115]
	LLE	Modified Folch, CHCl_3_ - MeOH - 0.15 M NaCl (2:1:0.8) + BHT	Lenses	DI-ESI-QqQ	[116]
	LLE	Modified Bligh and Dyer, CHCl_3_ - MeOH - 0.1N HCl (1:1:1)	Human cells (2 X 10^6^)	UPLC-ESI-QTOF	[117,118]
	µChip-SPE	Lysozyme in 20 mM Tris-HCl (pH 7.5)/Silica beads/MeOH	Bacterial cells (10 µL)	nanoESI-QTOF	[119]
	OPE	EtOH - MTBE - DCM (7:1.5:1.5)	Size-fractionated serum (20 µL)	UPLC-ESI-QTRAP	[35]
	LLE	Bligh and Dyer	Algae tissue (30 L culture)	UPLC-ESI-QTOF	[111]
***Glycerophosphoserines***					
	OPE	ACN	Plasma (20 µL)	UPLC-ESI-QTRAP	[113]
	LLE	CH_2_Cl_2_ - MeOH (2:1)	Cytosol (100 µL)	UPLC-ESI-QTRAP	[115]
	LLE	Bligh and Dyer	Human cells (0.4 × 10^6^)	ESI-QTRAP	[120]
	LLE	Modified Folch, CHCl_3_ - MeOH - 0.15 M NaCl (2:1:0.8) + BHT	Lenses	ESI-QqQ	[116]
	µChip-SPE	Lysozyme in 20 mM Tris-HCl (pH 7.5)/Silica beads/MeOH	Bacterial cells (10µL)	nanoESI-QTOF	[119]
***Glycerophosphoglycerols***					
	OPE	ACN	Plasma (20 µL)	UPLC-ESI-QTRAP	[113]
	LLE	Modified Bligh and Dyer, CHCl_3_ - MeOH - 0.1N HCl (1:1:1)	Human cells (0.4 × 10^6^)	UPLC-ESI-QTOF	[117,118]
	µChip-SPE	Lysozyme in 20 mM Tris-HCl (pH 7.5)/Silica beads/MeOH	Bacterial cells (10 µL)	nanoESI-QTOF	[119]
	LLE	Modified Bligh and Dyer, CHCl_3_ - MeOH - H_2_O (1:1:0.8)	Bacterial cells (OD: 0.3, 0.5, 0.8, 1.3)	UPLC-ESI-QqQ	[106]
	LLE	Bligh and Dyer	Algae tissue (30 L culture)	UPLC-ESI-QTOF	[111]
***Glycerophosphoinositols***					
	OPE	ACN	Plasma (20 µL)	UPLC-ESI-QTRAP	[113]
	LLE	CH_2_Cl_2_ - MeOH (2:1)	Cytosol (100 µL)	UPLC-ESI-QTRAP	[115]
	LLE	Modified Bligh and Dyer, CHCl_3_ - MeOH - 0.1N HCl (1:1:1)	Human cells (2 × 10^6^)	UPLC-ESI-QTOF	[117,118]
	µChip-SPE	Lysozyme in 20 mM Tris-HCl (pH 7.5)/Silica beads/MeOH	Bacteria Cells (10 µL)	nanoESI-QTOF	[119]
	OPE	EtOH - MTBE - DCM (7:1.5:1.5)	Size-fractionated serum (20 µL)	UPLC-ESI-QTRAP	[35]
***Glycerophosphates***					
	OPE	ACN	Plasma (20 µL)	UPLC-ESI-QTRAP	[113]
	µChip-SPE	Lysozyme in 20 mM Tris-HCl (pH 7.5)/Silica beads/MeOH	Bacterial cells (10µL)	nanoESI-QTOF	[119]
***Glycerophosphoglycerophosphoglycerols***					
	LLE	Modified Bligh and Dyer, CHCl_3_ - MeOH - H_2_O (1:1:0.8)	Bacterial cells (OD: 0.3, 0.5, 0.8, 1.3)	HPLC-ESI-QTOF	[106]
***Oxidized glycerophospholipids***					
	µHP-SPE	MeOH/C18 spin column/MeOH - 0.2% FA (82:18)	Plasma (20 µL)	HPLC-ESI-QTRAP	[121]
**Sphingolipids**					
***Sphingoid bases***					
	LLE	Modified Bligh and Dyer, CHCl_3_ - MeOH -H_2_O (1.8:2:0.8) + 0.1% TFA	Plasma (50 µL)	UHPLC-ESI-QqQ	[122]
	LLE	Bligh and Dyer, CHCl_3_ - MeOH - H_2_O (1:2:0.8)	B Cells (80 µL cell suspension)	HPLC-ESI-QTRAP	[123]
	LLE	MTBE - MeOH - H_2_O (3:0.9:0.8)	Plasma-Red blood cells (50 µL)	UHPLC-ESI-QqQ	[124]
	LLE + Transesterification	0.25M MeONa in MeOH - MTBE - H_2_O (1.3:4:1) + AcOH (pH 7)	Serum (40 µL)	UHPLC-ESI-QqQ	[125]
	LLE + Sap.	CH_2_Cl_2_ - MeOH - KOH 1M in MeOH (1:2:0.3)	Cells (10^6^)-Tissue (1–10 mg)	LC-MS/MS techniques	[126]
	LLE	CH _2_Cl_2_ - MeOH (1:1) with 0.25% DEA	Plasma (25 µL)	HPLC-ESI-QqQ	[127]
	LLE	ButOH - 40 mM Na_2_HPO_4_ + CA (pH4) (1:1)	Plasma (75 µL)	HPLC-ESI-QTRAP	[128]
***Ceramides***					
	LLE	Modified Bligh and Dyer, CHCl_3_ - MeOH -H_2_O (1.8:2:0.8) + 0.1% TFA	Plasma (50 µL)	UHPLC-ESI-QqQ	[122]
	LLE	Bligh and Dyer, CHCl_3_ - MeOH - H_2_O (1:2:0.8)	B Cells (80 µL cell suspension)	HPLC-ESI-QTRAP	[123]
	LLE + Sap.	CHCl_3_ - MeOH - 1M KOH in MeOH (1:2:0.3)	Cells (10^6^)-Tissue (1–10 mg)	LC-MS/MS techniques	[126]
	LLE	CH_2_Cl_2_ - MeOH (1:1) with 0.25% DEA	Plasma (25 µL)	HPLC-ESI-QqQ	[127]
	LLE + Transesterification	0.25M MeONa in MeOH - MTBE - H_2_O (1.3:4:1) + AcOH (pH 7)	Serum (40 µL)	UHPLC-ESI-QqQ	[125]
	LLE	ButOH - 40 mM Na_2_HPO_4_ + CA (pH4) (1:1)	Plasma (75 µl)	HPLC-ESI-QTRAP	[128]
	1. OPE, 2. SPE	1.MeOH, 2.Hex-*i*PrOH (11:1)/aminopropyl silica cartridges/Hex-CHCl_3_-MeOH (80:10:10)	Skin (Three patches)	UHPLC-ESI-QTOF	[129]
***Phosphosphingolipids***					
	LLE	Modified Bligh and Dyer, CHCl_3_ - MeOH -H_2_O (1.8:2:0.8) + 0.1% TFA	Plasma (50 µL)	UHPLC-ESI-QqQ	[122]
	LLE	CHCl_3_ - MeOH - 1M KOH in MeOH (1:2:0.3)	Cells (10^6^)-Tissue (1–10 mg)	LC-MS/MS techniques	[126]
	LLE + Transesterification	0.25M MeONa in MeOH - MTBE - H_2_O (1.3:4:1) + AcOH (pH 7)	Serum (40 µL)	UHPLC-ESI-QqQ	[125]
	OPE	MeOH	Whole blood (15 µL on DBS)	UHPLC-ESI-TOF	[130]
	OPE	AbsoluteIDQ p150 Kit	Plasma (10 µL)	FIA-ESI-QTRAP	[112]
***Neutral glycosphingolipids***					
	LLE	Bligh and Dyer, CHCl_3_ - MeOH - H_2_O (1:2:0.8)	B Cells (80 µL cell suspension)	HPLC-ESI-QTRAP	[123]
	LLE	CHCl_3_ - MeOH - 1M KOH in MeOH (1:2:0.3)	Cells (10^6^)-Tissue (1–10 mg)	LC-MS/MS techniques	[126]
	LLE	ButOH - 40 mM Na_2_HPO_4_ + CA (pH 4) (1:1)	Plasma (75 µL)	HPLC-ESI-QTRAP	[128]
	LLE	MTBE - MeOH - H_2_O (3:0.9:0.8)	Plasma-Red blood cells (50 µL)	UHPLC-ESI-QqQ	[124]
***Acidic glycosphingolipids***					
	LLE	CHCl_3_ - MeOH - H_2_O (0.8:1:1) + Deriv. with PAEA and DMTMM to aq. phase	Plasma (20 µL)	UHPLC-ESI-QTRAP	[131]
	1. LLE, 2. SPE	1. Modified Folch, CHCl_3_ - MeOH -H_2_O (2:1:0.6) 2.C18/MeOH to aq. phase	Plasma (200 µL)-Human tissues (25 mg)	HPLC-ESI-QTOF	[132]
	LLE	CH_2_Cl_2_ - MeOH (1:1) with 0.25% DEA	Plasma (25 µL)	HPLC-ESI-QqQ	[127]
**Sterol lipids**					
***Sterols***					
	SPE	Hex - Et_2_O (99:1)/NP silica cartridge/Hex - Et_2_O (99:1)	Sunflower oils (200 mg)	ESI-QTRAP-QqQ	[133]
	SPE	*i*PrOH/Polymeric RP/MeOH - 0.02% FA (10:90)	Plasma-Serum-CSF (100 µL)	HPLC-ESI-QTRAP	[134]
	LLE	Bligh and Dyer like method + Deriv. with AcCl - CHCl_3_ (1:5)	Ocular Tissue	HPLC-ESI-QTrap-Orbitrap	[135]
	1. OPE + Esterif. 2. LLE	1. *i*PrOH 70% + 5M NaOH (1M HCl), 2. *i*Oct - Reaction Mix. (2:1) + Deriv. with MSTFA	Feces (2 g)	GC-QqQ	[136]
	1. OPE + Esterif. 2. LLE	1. *i*PrOH 70%+5M NaOH (1M HCl), 2. *i*Oct-Reaction Mix.(2:1)+Deriv. with DMG+DMAP	Feces (2 g)	UHPL-HESI-Q-Orbitrap	[137]
	LLE	Hex - *i*PrOH - 0.47M Na_2_SO_4_ (2:3:1.5) + BHT	Atherosclerotic plaques (10 mg)	HPLC-APCI-QqQ	[138]
	1. OPE, 2. LLE	1. 50 mM Tris-HCl (pH 7.5), 150 mM NaCl and 2mM EGTA, 2. Modified Bligh and Dyer, CHCl_3_ - MeOH - Sample (1:1:0.9)	Silkworm tissues	HPLC-ESI-QqQ	[139]
***Steroids***					
	LLE	MTBE	Serum (100 µl)	HPLC-ESI-QTRAP	[140]
	SLE	Acetate buffer (pH 5.2)/Diat. Earth/CH_2_Cl_2_ + Deriv. with MSTFA - NH_4_I − DTE (500:4:2)	Serum (100 µl)	GC-QqQ	[141]
	SPE	5% H_3_PO_4_/Polymeric RP/MeOH	Plasma (100 µL)	HPLC-ESI-TripleTOF	[142]
***Bile acids and derivatives***					
	OPE	Methanol	Rat serum (10 μL)	UPLC-ESI-QTRAP/QTOF	[143]
	A. OPE or D. LLE	A. ACN; D: ACN - 400 g/L NH_4_SO_4_ - H_2_O (1:1:0.35)	Serum (100 µL)	HPLC-ESI-QqQ	[144]
	PD-SPE	Ostro 96 well plates/cold ACN with 1% of FA	Plasma-Serum (100 µL)	UHPLC-ESI-QqQ	[145]
	SPE	*i*PrOH/Polymeric RP/MeOH - 0.02% FA (10:90)	Plasma-Serum-CSF (100 µL)	HPLC-ESI-QTRAP	[134]
	OPE	iPrOH	Feces (1 g)	HPLC-ESI-QqQ	[146]
	1. OPE, 2. SPE	1. Saline solution - (60 °C) Ethanol (1:1); 2. Extract - H2O (1:10)/Polymeric RP/MeOH	Rat brain tissue (1.5–1.8 g)	HPLC-ESI-QqQ	[147]
**Prenol lipids**					
***Isoprenoids***					
	LLE	Et_2_O - PBS (pH = 7.4) - EtOH (1:1:0.2)	Feces (0.3–0.5 g)	HPLC-DAD	[148]
	OPE	ACN - MeOH - H_2_O (2:1:1)	Natural rubber (1.5 g)	HPLC-ESI-QqQ	[149]
	OPE	Methanol - H_2_O (1:1)	Bacterial cells (1.5 mL, OD: 5)	HPLC-ESI-TOF	[150]
	OPE	Methanol - H_2_O (1:3)	Bacterial supernatant (1.5 mL)	HPLC-ESI-TOF	[150]
	OPE	*i*PrOH - 100 mM NH_4_HCO_3_ (pH 7.4) (1:1)	Human cells	UPLC-ESI-QqQ	[151]
	SPE	2% FA/Polymeric RP/Hex - *i*PrOH - NH_4_OH (12:7:1)	Plasma (300 µL)	UPLC-ESI-QqQ	[151]
***Quinones and hydroquinones***					
	LLE	Saturated K_2_CO_3_ - CH_2_Cl_2_ (1:1)	Urine (Rats: 2 mL Human: 10 mL)	GC-Q	[152]
	LLE	CHCl_3_ - MeOH (3:7) - cold 10% NaCl + BHT	Bacterial cells (10–50 mg)	HPLC-APCI-QTRAP	[153]
**Saccharolipids**					
***Acylaminosugars***					
	1. LLE, 2. LLE	1. 45% Phenol - H_2_O; 2. CHCl_3_ - MeOH - H_2_O (2:1:3) + Deriv. aq phase.	Bacterial cells (2 g/mL)	GC-Q	[154]
	LLE	50% Phenol - H_2_O	Acetone-dried bacteria	UHPLC-ESI-QTOF	[155]
	SPE	Genlantis SoluLyse detergent/AEC/5% NH_4_OH in MeOH	Supernatant cell lysate (2×900 μL)	UPLC-ESI-QTRAP	[156]
**Polyketides**					
***Linear polyketides***					
	LLE	EtOAc	Bacterial supernatant (10 mL)	HPLC-ESI-LTQ-Orbitrap	[157]
	OPE	Acetone - MeOH (1:1)	Bacterial cells (10 mL culture)	HPLC-ESI-LTQ-Orbitrap	[157]
	OPE	MeOH	Bacterial supernatant (10 mL)	UPLC-ESI-QTOF	[158]
***Aromatic polyketides***					
	LLE	EtOAc + 1% HCl	Enzymatic mixture (500 µL)	HPLC-ESI-LTQ-Orbitrap	[159]
***Flavonoids***					
	OPE	70% MeOH + 3% FA	Digested cooked flour (1 mL)	UHPLC-HESI-QTRAP	[160]
	LLE	EtOAc	Blood-Tissue (50 µL)	UFLC-ESI-QqQ	[161,162]
***Polyether antibiotics***					
	1. OPE, 2. OPE, 3. LLE, 4. SPE	1. MeOH; 2. 80% PrOH; 3. EtOAc - H_2_O (1:1); 4. aq. phase/C18/80% PrOH	Microalga cells pellet (125–150 mL culture)	UPLC-nanoESI-QTOF	[163]
***Annonaceae acetogenins***					
	1. OPE, 2. LLE	1. Acetone; 2. CH_2_Cl_2_ - H_2_O (1:1)	Avocado mesocarp (2g)-cotyledons (1g)	HPLC-ESI-TOF	[164]

AcCl: Acetyl chloride, Ace: Acetone, ACN: Acetonitrile, AcOH: Acetic acid, AEC: Anion exchange cartridge, AMPP: N-[4-(aminomethyl)phenyl]pyridinium, APCI: Atmospheric pressure chemical ionization, API: Atmospheric pressure ionization, aq.: aqueous, BHT: Butylated hydroxytoluene, BHT: Butylhydroxytoluene, BSTFA: N,O-Bis(trimethylsilyl)trifluoroacetamide, ButOH: n-Butanol, CA: Citric acid, Conc.: Concentration, CSF: cerebrospinal fluid, DAD: Diode Array Detection, DEA: Diethylamine, Deriv.: Derivatization, Diat.: Diatomaceous, DI: direct-infusion, DMAP: N,N-dimethylpyridin-4-amine, DMED: N,N-dimethylethylenediamine, DMG: N,N-dimethylglycine, DMTMM: 4-(4, 6-Dimethoxy-1,3,5-triazin-2-yl)-4-methylmorpholinium chloride, DnsHz: Dansylhydrazine, DTE: Dithioerythritol, EDTA: Ethylenediaminetetraacetic acid, EGTA: ethylene glycol-bis(β-aminoethyl ether)-N,N,N′,N′-tetraacetic acid, eq.: Equivalent, Et_2_O: Diethyl ether, ESI: Electrospray ionization, EtOAc: Ethyl acetate, FA: Formic acid, FIA: Flow Injection Analysis, FT-ICR MS: Fourier transform ion cyclotron resonance mass spectrometry, HESI: Heated electrospray ionization mode, Hex: n-Hexane, iOct: 2,2,4-trimethylpentane (isooctane), iPrOH: 2-propanol, LLE: Liquid–liquid Extraction, LTQ: Linear Trap Quadrupole, MeONa: Sodium methoxide, MF: Methyl formate, MSTFA: N-Methyl-N-trimethylsilyl-trifluoracetamide, MTBE: Methyl tert-butyl ether, nanoESI: nano electrospray ionization, NH_4_OAc: Ammonium acetate, NP: Normal phase, NPB: 3-nitrophenylboronic acid, OPE: One Phase Extraction, PAEA: 2-(2-Pyridilamino)-ethylamine, PBS: Phosphate Buffered Saline, PC: Protein content, PD-SPE: Phospholipid depletion solid phase extraction, PGWAT: Perigonadal white adipose tissue, PP: Potassium Phosphate, PrOH: 1-propanol, Q: Quadrupole, RP: Reverse Phase, SLE: Supported Liquid Extraction, SPE: Solid Phase Extraction, TFA: Trifluoroacetic acid, TOF: Time of flight, Tris-HCl: Tris(hydroxymethyl)aminomethane hydrochloride, UFLC: Ultra Fast Liquid Chromatography, µChip-SPE: Microchip-based SPE, µHP-SPE: micro-preparative high-performance solid-phase extraction. References were selected for studies conducted during the 2011–2020 period.
